# Effectiveness of Online Training on Cardiopulmonary Resuscitation and Automated External Defibrillator Use in Simulations With Rural Populations: Quasi-Experimental Community Intervention Study

**DOI:** 10.2196/80359

**Published:** 2026-05-22

**Authors:** Elena Taverna-Llauradó, Sara Martinez-Torres, Alba Roca-Biosca, Meritxell Pallejà-Millán, Francisco Martín-Luján, Cristina Rey-Reñones

**Affiliations:** 1 Primary Care Unit Camp de Tarragona, Institut Català de la Salut, Reus, Spain; 2 Primary Healthcare Research Support Unit Camp de Tarragona, Institut Universitari d'Investigació en Atenció Primària Jordi Gol, Reus, Spain; 3 TIC-AP Study Group, Primary Healthcare Research Support Unit Camp de Tarragona, Institut Universitari d'Investigació en Atenció Primària Jordi Gol, Reus, Catalunya, Spain; 4 Universitat Oberta de Catalunya, Barcelona, Spain; 5 Nursing Department, Universitat Rovira i Virgili, Tarragona, Spain; 6 School of Medicine and Health Sciences, Universitat Rovira i Virgili, C/St Llorenç 21, Reus, 43201, Spain, 1 977778515

**Keywords:** out-of-hospital cardiac arrest, cardiopulmonary resuscitation, automated external defibrillator, emergency medical services, primary care, rural medicine

## Abstract

**Background:**

Sudden death due to cardiorespiratory arrest has a high mortality rate and often occurs outside hospital settings. Prompt initiation of cardiopulmonary resuscitation (CPR) by bystanders, along with the use of an automated external defibrillator (AED), has been shown to double survival rates. Given the challenges of ensuring timely emergency response in rural areas, implementing basic CPR training programs can help improve survival outcomes.

**Objective:**

This study aimed to evaluate the effectiveness of online CPR-AED training delivered to participants from a rural area of Camp de Tarragona.

**Methods:**

This quasi-experimental study consisted of 2 phases: phase 1, evaluation of the effectiveness of online training on CPR-AED knowledge (with pretest and posttest assessments), and phase 2, evaluation of the effectiveness of online training on CPR-AED maneuvers through simulation at 1 month and 6 months after online training. The sample of the study comprised residents in a rural area of Tarragona, Spain. A descriptive statistical analysis of the study population was conducted. For quantitative data with a nonnormal distribution, the median and IQR were presented. Categorical data were described as frequencies and percentages. A bivariate analysis was performed to compare the pretraining and posttraining quantitative variables using the Student 2-tailed *t* test.

**Results:**

In total, 55 participants were included in the study. Of these, 74.5% (n=55) were women, the mean age was 41.5 (SD 9.1) years, and 94.5% (n=55) were employed. Overall, 52 participants completed the online training. The median time required to complete the course was 261.5 (IQR 935; range 125-327) minutes. In total, 51 participants took part in the first practical simulation, which was conducted 1 month after the theoretical training. The mean score obtained in this first simulation was 7.5 out of 10. Six months after the theoretical training, 46 participants completed a second simulation.

**Conclusions:**

Online CPR-AED training is effective in improving CPR-AED knowledge and skills in a rural population in the short and medium term.

## Introduction

Out-of-hospital cardiac arrest (OHCA) remains a major cause of mortality worldwide, with survival critically dependent on early recognition, prompt initiation of cardiopulmonary resuscitation (CPR), and rapid use of an automated external defibrillator (AED). Despite sustained public health initiatives, recent epidemiological data indicate that bystander CPR and AED deployment remain suboptimal in many regions, particularly in rural or geographically dispersed populations [1]. In Europe, OHCA represents a substantial public health burden, with an estimated annual incidence ranging from 53 cases per 100,000 inhabitants in Austria to 166 per 100,000 in Bosnia and Herzegovina [2].

Although advances in prehospital and emergency care have improved outcomes, overall survival remains limited. The EuReCa TWO registry, encompassing resuscitation attempts in 28 European countries, reported survival to hospital discharge in only 8% of patients [2].

In Spain, national registry data from 2022 documented an OHCA incidence of 24.2 cases per 100,000 inhabitants, with marked regional variation, with Catalonia reporting one of the highest incidence rates at 35.2 cases per 100,000. Overall survival to hospital discharge was 11.5%. These outcomes remain strongly associated with the timeliness and quality of bystander intervention, particularly CPR provision and AED accessibility. The “chain of survival”—early recognition, timely bystander CPR, rapid defibrillation, advanced life support, and postresuscitation care—continues to be central to improving OHCA outcomes [3].

Each minute of delay in chest compressions reduces survival by approximately 10%, whereas defibrillation within 3 to 5 minutes for shockable rhythms may increase survival to as much as 30% [4]. However, bystander CPR rates vary widely, from 26% in some regions to approximately 69% in communities with comprehensive public training programs.

Rural populations face distinct barriers to emergency response education, including limited access to certified instructors, long travel distances to training sites, and fewer opportunities for refresher courses [5]. These structural constraints likely contribute to lower CPR training rates and reduced AED awareness compared with urban communities, potentially widening disparities in OHCA outcomes [1]. In such settings, early intervention often depends on lay bystanders before the arrival of emergency medical services (EMS) [6-8].

Hands-only CPR has been demonstrated to be as effective as conventional CPR for most adult cardiac arrests, lowering barriers for lay responders and facilitating dissemination through community-based programs [6]. Nonetheless, substantial inequities persist between urban and rural areas. In rural contexts, EMS response times frequently exceed 10 to 15 minutes, AED availability is limited, and community CPR training coverage is lower. These challenges are often compounded by socioeconomic disadvantage, with underserved populations less likely to receive bystander CPR or early defibrillation [6].

Disparities in early resuscitation are particularly concerning in pediatric OHCA, where rapid intervention is essential. Globally, the annual incidence among children younger than 12 years ranges from 8 to 20 cases per 100,000 [9]. However, most schoolteachers report insufficient CPR training and limited confidence in performing or teaching resuscitation techniques. Although hands-only CPR is generally preferred by educators, overall preparedness remains inadequate [10].

Therefore, training schoolteachers in CPR may have a multiplier effect, enabling them to respond effectively in school environments while simultaneously transmitting life-saving skills to students and fostering a broader culture of preparedness within the community [11]. This approach is especially relevant in rural regions, where access to EMS is constrained and scalable digital solutions—such as online CPR courses—may represent practical and cost-effective alternatives.

Although traditional face-to-face CPR-AED training has proven effective, the COVID-19 pandemic accelerated the adoption of digital health education modalities, including blended and fully online programs. Emerging evidence suggests that online or hybrid CPR instruction combined with simulation-based practice can improve short-term knowledge retention and psychomotor skills, although findings remain heterogeneous and dependent on instructional design, learner characteristics, and assessment strategies [12].

Simulation-based assessment is increasingly used to evaluate CPR competence in standardized environments, enabling objective measurement of procedural accuracy, timing, and checklist-based performance [12,13]. However, improvements observed in simulated contexts should be interpreted cautiously, as such performance may not fully translate to real-world behavior during actual emergencies.

Despite growing interest in remote and community-based CPR education, evidence specifically examining online CPR-AED training for rural lay populations remains limited [1]. Most studies have focused on urban cohorts, health science students, or workplace settings, leaving an important knowledge gap regarding the feasibility and potential impact of digital training strategies in geographically isolated communities [13].

To address this gap, this study evaluates changes in theoretical knowledge and simulated CPR-AED performance among rural community participants before and after completion of an online training program supplemented by in-person simulation practice. Given the quasi-experimental pre-post design and the absence of a concurrent control group, the analysis focuses on observed improvements rather than causal inference.

## Methods

### Study Design, Setting, and Recruitment

This study was designed as a quasi-experimental community intervention without a control group. It was structured in two sequential phases:

- Phase 1: evaluation of the effectiveness of online training on CPR-AED theoretical knowledge
- Phase 2: evaluation of the effectiveness of online training on CPR-AED practical skills through in-person, simulation-based assessment

The study was carried out in 2 primary health care centers located in the municipalities of Borges del Camp and Cornudella de Montsant (province of Tarragona, Catalonia, Spain). The reference population consisted of residents from 3 rural municipalities in Catalonia.

A systematic community-based recruitment strategy was implemented. First, the research team contacted local authorities and institutional representatives to present the objectives and procedures of the study and to request collaboration. The study was subsequently disseminated through municipal associations, schools, and secondary education institutes, as well as through community notice boards and local communication channels. Informational meetings were organized when it was feasible to explain the study and resolve questions from potential participants.

Interested individuals contacted the research team or the primary health care centers directly. After receiving detailed information about the study, those willing to participate provided informed consent and were consecutively enrolled until the target sample size was reached. Recruitment procedures were identical across the participating municipalities to ensure consistency.

### Participant Selection

Participants were selected based on specific eligibility criteria.

The inclusion criteria were as follows:

- Residence and/or employment in the Priorat or Baix Camp regions
- Age ≥18 years
- Access to the internet and compatible devices to complete the online training

The exclusion criteria were as follows:

- Presence of a language barrier (limited or no comprehension of Catalan)
- Presence of any disability preventing participation in the course and/or performance of CPR and AED procedures
- Anticipated inability to attend simulation sessions for evaluation

Prior CPR-AED training was not considered as an exclusion criterion.

### Description of Intervention

The intervention consisted of an online training program based on the European Resuscitation Council (ERC) guidelines, as described in detail in the study protocol [10,14].

The primary objective of the training was to provide both theoretical and practical knowledge regarding emergency situations and the use of an AED.

Participants interested in enrolling were required to register on a Moodle platform, where all training materials were hosted. The course included interactive modules, audiovisual resources, and assessments to ensure the acquisition of both theoretical and practical competencies.

### Evaluation of Intervention

#### Phase 1: Assessment of Knowledge Acquisition Following Online CPR-AED Training

To evaluate the effectiveness of the online training program, participants completed 2 standardized knowledge assessments: a pretraining test to establish baseline knowledge and a posttraining test to measure knowledge gained after course completion.

The assessment instrument was developed in accordance with the guidelines of the Canadian Council on Resuscitation and the ERC and consisted of 10 multiple-choice questions. Participants were required to achieve a perfect score (10/10) to pass. An unlimited number of attempts was permitted, and results were categorized dichotomously as pass or fail. Only participants who successfully completed the posttraining assessment were eligible to participate in the subsequent phase of the study.

#### Phase 2: Assessment of Practical Skill Retention Over Time

Participants who consented to continue in the study were invited to perform a simulated CPR-AED scenario at 2 follow-up intervals: 1 month (short term) and 6 months (medium term) after completing the online training.

During these sessions, participants were exposed to a standardized cardiac arrest scenario requiring the application of CPR and AED skills. Each simulation was independently assessed by 2 trained observers using a structured checklist to assess performance and retention of critical skills. The items evaluated in the checklist were (1) safe approach, (2) check response, (3) open the airway, (4) check for spontaneous breathing, (5) request external assistance, (6) ventilation-compressions ratio, (7) quality of chest compressions, (8) patch placement, (9) AED use, and (10) immediate compressions. At the end of the simulation, the participants were informed of the mistakes they made and, when applicable, how they should have proceeded.

### Outcome Measures

#### Effectiveness of Online Training on CPR-AED Knowledge

The primary outcome measure of effectiveness was the difference in scores between the pretraining assessment and the first attempt of the posttraining assessment at the end of the course. Secondary variables included the number of attempts required to achieve a perfect score and the score obtained on the first posttraining attempt.

#### Effectiveness of Online Training on CPR-AED Performance in a Simulated Environment

In phase 2, the primary outcome was the performance score obtained during the in-person simulation. Success in the simulation was defined as the correct execution of at least 8 of 10 key actions listed on a standardized evaluation checklist [15,16].

To evaluate the effectiveness of the training in the simulation, the necessary sample size was calculated using the statistical software GRANMO (version 7.11) using the option of averages observed with respect to a reference sample. Accepting an α risk of .05 and a β risk of .2 for a bilateral contrast, 52 participants were needed to detect a difference of ≥0.5 points in the face-to-face evaluation of the online training, assuming a common SD of 1.07. The number of participants was increased by 30% to compensate for potential losses to follow-up [14].

### Statistical Analyses

A descriptive analysis of the study population was conducted. Quantitative variables following a normal distribution are expressed as mean (SD), whereas nonnormally distributed variables are presented as median (IQR). Normality was assessed using the Kolmogorov-Smirnov test. Categorical variables are reported as frequencies and percentages.

Bivariate analyses were performed to compare pretraining and posttraining quantitative variables using the Student 2-tailed *t* test.

### Ethical Considerations

The study was conducted in accordance with the principles of the Declaration of Helsinki and the guidelines of good clinical practice. The protocol was approved by the clinical research ethics committee of the Primary Care Research Institute IDIAP Jordi Gol (23/081-P) on June 15, 2023.

Data confidentiality was ensured in compliance with Spanish legislation on personal data protection (Ley Orgánica 03/2018 of December 5, on the Protection of Personal Data and Guarantee of Digital Rights) [17]. Participants received an information sheet explaining that their participation was voluntary, anonymous, and confidential and that they could withdraw from the study at any time prior to data verification. All participants were given the opportunity to ask questions and receive clarification from the research team before enrollment. Verbal and written informed consent for participation and audio recording were obtained from all participants.

Each participant was assigned a unique identification code upon inclusion. Study data were recorded using a purpose-designed electronic questionnaire and stored on a secure digital platform accessible only through the corporate intranet of the Catalan Health Institute in Tarragona. Access was password-protected and restricted to authorized investigators responsible for data entry. The dataset is not publicly available; however, access may be granted upon reasonable request to the principal investigator and with approval from the IDIAP Jordi Gol Research Ethics Committee.

Participants did not receive any financial compensation for their involvement in the study. Study findings will be disseminated to participating centers and research teams and shared with the scientific community through peer-reviewed publications and conference presentations. Dissemination in nonspecialist settings is also planned.

## Results

### Sociodemographic Characteristics of the Population

**Figure 1.**
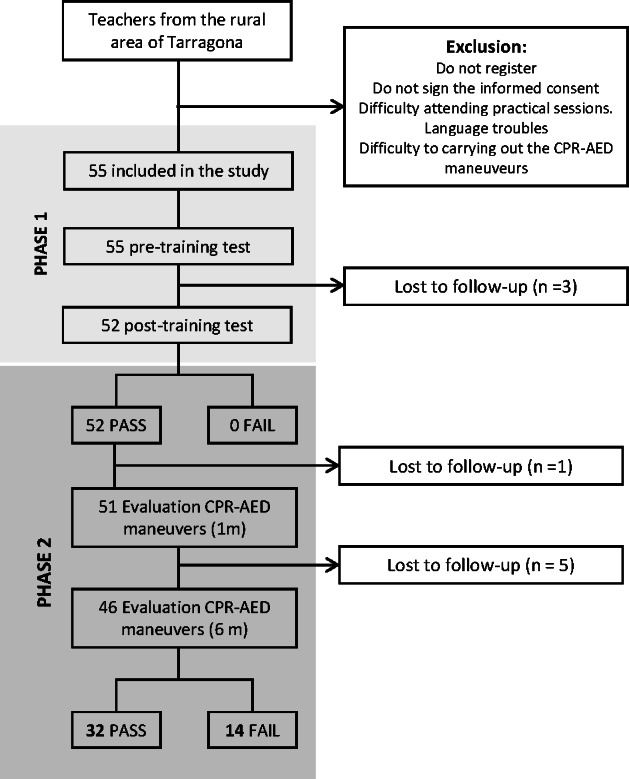
Flowchart of participant recruitment and inclusion in a quasi-experimental community intervention study evaluating the effectiveness of an online CPR-AED training program among rural community residents in Catalonia, Spain. The diagram shows the stages of eligibility assessment, enrollment, allocation to phase 1 (online theoretical training), participation in phase 2 (in-person simulation-based skills assessment), and final analysis. CPR-AED: cardiopulmonary resuscitation–automated external defibrillator.

In total, 55 participants were included in the study (Figure 1). Of these, 74.5% (n=55) were women, 94.5% (n=55) belonged to the active working population, and 60% (n=33) held a university degree. Slightly more than half (n=32, 58.2%) of the participants had received previous training related to the topic under study. Overall, 7.3% (n=4) had previously witnessed a cardiac arrest; however, none reported knowing how to respond appropriately. Most participants resided in rural areas of the Baix Camp region. The mean age of the participants was 41.5 years. A summary of the main characteristics of the study population is presented in Table 1.

**Table 1.** Main sociodemographic characteristics of the study population (N=55).

Variables	Participants, n (%)
Sex	
Female	41 (74.5)
Male	13 (23.6)
Nonbinary	1 (1.8)
Employment status	
Student	2 (3.6)
Unemployed	1 (1.8)
Employed	52 (94.5)
Educational level	
University diploma	33 (60)
Bachelor’s degree or higher	17 (30.9)
Nonqualified	1 (1.8)
Skilled nonmanual	3 (5.5)
Other	1 (1.8)
Participants with previous training	32 (58.2)
Participants who had witnessed an arrest	4 (7.3)
Participants who did not know how to respond to the arrest	4 (100)
Place of residence: rural	39 (70.9)
Region	
Baix Camp	41 (74.5)
Priorat	6 (10.9)
Alt Camp	2 (3.6)
Tarragonès	5 (9.1)
Ribera d’Ebre	1 (1.8)

### Phase 1: Evaluation of the Effectiveness of Online Training on CPR-AED Theoretical Knowledge

Of the 55 individuals enrolled in the study, 52 (94.5%) participants completed the online training. The median time required to complete the course was 201.5 minutes, with an IQR of 935 minutes.

The median number of pretest attempts was 1 (IQR 0). The mean pretest score was 6.72 (SD 2.06) points out of 10. The median number of posttest attempts was 2 (IQR 1), with a mean posttest score of 7.77 (SD 1.10) points out of 10.

The mean difference between pretraining and posttraining scores was 1.05 (SD 2.06) points, which was statistically significant (*P*=.01). These data are presented in Table 2.

**Table 2.** Main variables analyzed from the online training.

Variables	Values
Time taken to complete the course (minutes), median (IQR)	201.5 (935)
Number of pretest attempts, median (IQR)	1 (0)
Pretest score (out of 10), mean (SD)	6.72 (2.06)
Number of posttest attempts, median (IQR)	2 (1)
Posttest score (out of 10), mean (SD)	7.77 (1.10)

### Phase 2: Evaluation of the Effectiveness of Online Training on CPR-AED Skills Through Simulation-Based Assessment

Of the 52 participants who completed the online training, 51 (98.1%) took part in the first practical simulation, which was carried out 1 month after the theoretical training. The average score obtained in this first simulation was 7.5 out of 10.

Six months after the theoretical training, 46 participants completed a second simulation. Among them, 42 achieved a passing score—defined as ≥8 points—representing 91.3% of those who participated in this second practical test. The average score was 9.0 out of 10. In contrast, 4 (8.7%) participants did not reach the minimum score to be considered competent, scoring ≤7 points.

A statistically significant improvement was observed between the first and final simulation scores, with a mean difference of 1.31 points (SD 2.05; *P*<.001), indicating a positive evolution in performance over time. Additionally, a paired-samples analysis comparing the scores from both simulations showed a significant increase in the second attempt, with an average difference of 0.89 points (*P*=.01), further supporting the sustained effect of the training over 6 months. The main results obtained in the simulation phases are shown in Table 3.

**Table 3.** Main results obtained in the simulation phases.

Simulation phases	First simulation (n=51), n (%)	Second simulation (n=46), n (%)
Safe approach	36 (70.6)	32 (69.6)
Check response	41 (80.4)	37 (80.4)
Open the airway	19 (37.2)	17 (37)
Check for spontaneous breathing	42 (82.4)	38 (82.6)
Request external assistance	31 (60.8)	33 (71.7)
Compressions-breathing relation	41 (80.4)	37 (80.4)
Quality of chest compressions	31 (60.8)	30 (65.2)
Patch placement	41 (80.4)	37 (80.4)
Automated external defibrillator use	40 (78.4)	36 (78.3)
Immediate compressions	44 (86.3)	39 (84.8)

An analysis of different variables revealed that, in the first simulation, there were no significant differences in scores based on participants’ gender, place of residence (rural or urban), prior experience witnessing a cardiac arrest, or having received previous CPR or AED training. These factors did not appear to influence initial practical performance. However, in the second simulation, a significant association was found between having previous training and achieving a passing score (*P*=.004), suggesting that prior exposure to resuscitation concepts may enhance long-term skill retention. No significant differences were observed in the second simulation based on sex, place of residence, or previous experience witnessing a cardiac arrest. The differences in simulation scores according to participant characteristics are presented in Table 4.

**Table 4.** Differences between the punctuation of the simulation according to different variables.

Variables	First simulation, n (%)			Second simulation, n (%)		
	Pass	Fail	*P* value	Pass	Fail	*P* value
Gender			.58			.21
Woman	17 (51.5)	16 (48.5)		24 (75)	8 (25)	
Man	6 (50)	6 (50)		8 (61.5)	5 (38.5)	
Nonbinary	0 (0)	1 (100)		0 (0)	1 (100)	
Participants with previous training	14 (58.3)	10 (41.7)	.24	22 (8.5)	5 (18.5)	.04
Participants who had witnessed an arrest	1 (25)	3 (75)	.29	3 (75)	1 (25)	.81
Place of residence: rural	18 (52.9)	16 (47.1)	.47	23 (67.6)	11 (32.4)	.63

## Discussion

This community-based study evaluated changes in theoretical knowledge and simulated CPR-AED performance following completion of an online training program supplemented with in-person simulation among lay participants—primarily primary and secondary schoolteachers—from rural areas in southern Catalonia.

Consistent with the study objectives, participants demonstrated significant improvements in posttraining knowledge scores and simulation performance compared with baseline. These findings suggest that a digitally delivered CPR-AED curriculum, when paired with structured hands-on practice, may enhance preparedness among non–health care professionals in geographically dispersed settings.

Out of the 55 individuals initially recruited, 52 completed the posttraining evaluation. Median knowledge scores increased from 6.72 (IQR 6-8.57) to 7.77 (IQR 7-8.67 out of 10), and participants required fewer attempts to complete the theoretical assessment, suggesting improved mastery of key resuscitation concepts. In addition, the improvement observed between the 2 simulated scenarios indicates retention of procedural skills, which is particularly relevant given that simulation assessments focused on core elements of basic life support and AED use. Although participants could repeat the test, the observed reductions in errors and increased procedural accuracy may reflect learning through feedback and iterative practice.

Unlike many community courses that focus solely on hands-only CPR, this program provided comprehensive basic life support training, including AED deployment. The online format offered logistical advantages and the potential to reach a broad audience, particularly in rural areas where access to face-to-face instruction is often limited.

All simulations were conducted individually at the participants’ workplaces using manikins and standardized cardiac arrest scenarios under supervision by certified instructors and observers, following the Catalan Council of Resuscitation guidelines derived from the ERC. The observed gains are consistent with prior studies demonstrating the feasibility and educational value of virtual or blended CPR instruction for lay populations [18-20].

However, the characteristics of the study sample warrant cautious interpretation. Approximately 70% of participants were women, 60% held a university degree, and approximately 30% resided in urban areas while working in rural schools. These demographic features may limit generalizability to the broader rural population and suggest that participants may have been more motivated or educationally advantaged than the general community.

Several limitations should be considered. First, the quasi-experimental pre-post design without a control group precludes causal inference regarding the effectiveness of the intervention. Second, although simulation-based assessments provide standardized and objective measures of performance, they may not fully reflect behavior during real-life cardiac arrest events.

Technical barriers also affected implementation, as internet connectivity was inconsistent in some rural locations, potentially limiting access to the online materials. Additionally, some substitute teachers who participated in the first simulation were unable to complete follow-up assessments because they were no longer employed at the same schools, resulting in attrition between testing phases.

Finally, the demographic composition of the sample and partial inclusion of participants residing in urban settings restrict external validity and should be taken into account when extrapolating these findings to other rural contexts.

This study suggests that online CPR-AED training supplemented by workplace-based simulation may represent a scalable approach to improving resuscitation preparedness among laypeople in rural communities. Beyond immediate educational gains, such programs have the potential to strengthen community emergency response capacity by establishing local networks of trained individuals, particularly in school environments where teachers can serve both as first responders and multipliers of knowledge.

Participants’ recommendations to integrate CPR education into primary and secondary school curricula, provide regular refresher training, and improve community awareness of public-access AEDs align with international calls for school-based resuscitation education as a cornerstone of population-wide preparedness. Therefore, future public health strategies should consider incorporating accessible digital training platforms alongside broader AED deployment to reduce disparities in OHCA outcomes, particularly in underserved rural areas.

Further research using controlled designs, longer follow-up periods, and more representative rural samples is needed to determine whether observed improvements in knowledge and simulated performance translate into sustained behavioral change and improved real-world clinical outcomes.

## Abbreviations

AED: automated external defibrillator
CPR: cardiopulmonary resuscitation
EMS: emergency medical services
ERC: European Resuscitation Council
OHCA: out-of-hospital cardiac arrest
